# Chemical composition, antioxidant activities and antibacterial activities of essential oil from *Erythrina caffra* Thunb. growing in South Africa

**DOI:** 10.1016/j.heliyon.2021.e07244

**Published:** 2021-06-07

**Authors:** Olubunmi A. Wintola, Aderonke A. Olajuyigbe, Anthony J. Afolayan, Roger M. Coopoosamy, Olufunmiso O. Olajuyigbe

**Affiliations:** a Medicinal Plants and Economic Development (MPED) Research Centre, Botany Department, University of Fort Hare, Alice Campus, South Africa; b Department of Microbiology, School of Science & Technology, Babcock University, PMB 4005, Ilisan-Remo, Ogun State, Nigeria; c Department of Nature Conservation, Faculty of Natural Sciences, Mangosuthu University of Technology, P.O.Box 12363, Jacobs, 4026, Durban, Kwa-Zulu Natal, South Africa

**Keywords:** Antioxidant, Antibacterial, Susceptibility, Bioactive compounds, Caryophyllene oxide, GC-MS analysis, Phytochemicals

## Abstract

**Introduction:**

Essential oils from plants are recognized as one of the most promising secondary metabolites for the development of cheap and safer drugs. While *Erythrina caffra* has been prominently used in folk medicine for the treatment of microbial infections, there is dearth of information on the pharmacological effectiveness and chemical composition of its essential oil. The study, therefore, aimed at identifying the chemical composition and biological activities of the essential oil of *Erythrina caffra.*

**Methods:**

In this study, the essential oil was extracted with all-glass Clevenger. The antioxidant activities of the essential oil and antibacterial susceptibility assay by agar well diffusion techniques were assessed while GC-MS analysis was performed to identify the chemical constituents of the essential oil.

**Results:**

The study showed that the radical scavenging activity of the essential oil increases as the concentration of the essential oil increases. All bacterial isolates were susceptible to essential oil with the exception of *Salmonella typhimurium* and *Pseudomonas aeruginosa* producing inhibition zones ranging between 22 ± 1.3 and 35 ± 2.1 mm in the susceptible isolates. The GC-MS chromatogram indicated there are 35 bioactive compounds in the essential oil and Caryophyllene oxide (53.54%), [1S-(1α,7α,8aβ)]-1,2,3,5,6,7,8,8a-octa-1 - hydro-1,8a-dimethyl-7-(1-methylethenyl)-Naphthalene (7.81%), Kauran-18-al (6.49%), 10,10-Dimethyl-2,6-dimethylenebicy clo[7.2.0]undecan-5.beta.-ol (5.83%), 10s,11s-Himachala-3(12),4-diene (4.51%), Caryophyllene (3.65%) and 1- Hexanol (3.31%) were the most prominent compounds.

**Conclusion:**

Excessive production of free radicals or reactive oxygen species (ROS) causes oxidative stress and disease. Oxidative stress resulting from imbalance between excessive generation of free radicals and inadequate antioxidant defense system has been linked to pathogenesis of many diseases. The essential oil of *E. caffra* stem bark extract possess antimicrobial and good antioxidant activities and its rich level of phytochemicals can be used as either dietary or complementary agents.

## 1 Introduction

Consequential to the desire to treat and cure diseases becoming the primary concerns of mankind, plants and herbs have been used indigenously to treat ailments over the centuries. Being readily assessable and cheap sources of pharmacologically active agents, plant-derived medicines have been part of traditional health care in many parts of the world for centuries. Essential oils from plants are recognized as one of the most promising secondary metabolites for the development of cheap and safer drugs [1]. Essential oils are of medicinal importance in herbal medicine [2, 3, 4]. In foods, toiletries, fine chemicals and pharmaceutical industries, essential oils, their fractions and isolated aromatic chemicals are valuable ingredients. They are utilized as such or in diluted forms in therapy or by the aromatherapy sector [5]. While medicinal plants play important roles as source of antimicrobial, antioxidant and inflammatory agents [5, 6], those from plants possess constituents that have pharmacological importance in the prevention and treatment of chronic diseases and infections.

*Erythrina* is a genus of flowering plants in the pea family Fabaceae [7] which are often cultivated as an ornamental and soil improvement tree to fix nitrogen for other tree crops [8]. This genus has been introduced and cultivated in India and Kenya [9] and over 130 species are distributed in tropical and subtropical regions of the world. Nine of these trees are found in southern Africa where the most common species are probably the *Erythrina caffra* and *Erythrina lysistemon* growing up to 30 m in height. The stem barks of *Erythrina* species have many physiologically active alkaloids [10]. Their alkaloids are attractive synthetic targets resulting from their use in indigenous medicine [11]. The prenylated flavonoids, prevalent in the stem and root bark [12, 13], has displayed a variety of biological activities. Traditionally, *Erythrina* genus has been prominently used in folk medicine [14, 15]. Mitscher *et al.* [16] and Burkill [17] indicated that many of these species have been used for the treatment of female infertility, inflammation, stomach pain, gonorrhea and other microbial infections and as tranquilizers against insomnia [18]. *Erythrina caffra,* the Coastal Coral Tree, native to southeastern Africa, is used for treating sores, tuberculosis, respiratory and wound infections, arthritis and toothache while the extracts of *E. senegalensis, E. velutina* and *E. mulungu* have demonstrated the presence of analgesic and anti-inflammatory effects [19]. However, while there are sufficient scientific reports confirming the ethnopharmacological importance of *E. caffra* [20], the chemical composition of its essential oil, its antioxidant and antibacterial properties remains vague and the pharmacological effectiveness needed to be investigated. Therefore, this study aimed at identifying the chemical composition of the essential oil of *Erythrina caffra* using gas chromatography-mass spectrometry (GC-MS) technique, evaluate its antioxidant and antibacterial potentials using standard procedures and to justify their possible multi-functional roles in folklore uses.

## 2 Materials and methods

### 2.1 Plant collection and preparation

The bark materials of *E. caffra* were collected from the plant growing within the University of Fort Hare campus in Alice, South Africa. The plant was authenticated in the Department of Botany and the voucher specimen (FO/Med 2014/01) was deposited in the Giffen's herbarium of the University. The study area lies at the latitude 30^o^ 00 to 34^o^15′S and longitudes 22^o^ 45′ to 30^o^ 15′E. It is bounded by the sea in the East and the drier Karoo (semi-desert vegetation) in the West. The elevation ranged from sea-level to approximately 2,200 m in the north and the vegetation is veld type. It is known as the Eastern Cape thorn veld [21]. The stem bark materials were properly washed, dried, pulverized and kept in a refrigerator at 4 °C until time for essential oil extraction.

### 2.2 Collection of plant materials and extraction of the essential oil

For the extraction of essential oil from the stem bark material, 250 g of the stem bark of the plant was hydro-distilled for 3 h in an all-glass Clevenger [22]. Heat was supplied to the heating mantle (50 °C) and the essential oil was extracted with 4 L of water for 3 h until essential oil was no more released. At the end of the distillation, the essential oil was collected, dried under anhydrous sodium sulphate and stored in sealed vials in the dark at 4 °C until used. The average percentage yield was 0.4%. The essential oil was later dissolved with appropriate vehicles for further *in vitro* bioassay activities.

### 2.3 Chemicals and reagents used

The chemicals used include 1,1-diphenyl-2-picrylhydrazyl (DPPH), 2,2′-azino-bis (3-ethylbenzthiazoline-6-sulphonic acid (ABTS), vanillin, butylated hydroxyl toluene (BHT), rutin, potassium persulphate, sodium nitroprusside (Na_2_[Fe(CN)_5_NO]2H_2_O), sulphanilic acid, glacial acetic acid (CH_3_COOH), gallic acid, tannic acid, ferric chloride (FeCl_2_), ascorbic acid, Folin-Ciocalteu reagent, sodium carbonate (Na_2_CO_3_), aluminum chloride (AlCl_3_), potassium acetate (CH_3_CO_2_K), potassium ferricyanide[K_3_Fe(CN)_6_], trichloroacetic acid (TCA), 2-thiobarbituric acid (TBA), hydrochloric acid (HCl), sodium chloride (NaCl), sodium dihydrogen phosphate (NaH_2_PO_4_), disodium hydrogen phosphate (Na_2_HPO_4_), dimethyl sulphoxide (DMSO), Mueller-Hinton Agar (MHA), Mueller-Hinton dextrose broth (MDB) and amoxicillin. These chemicals were purchased from Merck and Sigma-Aldrich, Gauteng, South Africa. All the chemicals used, in this study, were of analytical grade.

### 2.4 Antioxidant assay

The antioxidant activities of the essential oil from the stem bark of *Erythrina caffra* were determined by using nitric oxide, reducing power, hydrogen peroxide, lipid peroxidation inhibitory assays, DPPH, and ABTS scavenging activity assays.

### 2.5 Nitric oxide scavenging activity assay

The nitric oxide radical scavenging activity was determined with method described by Oyedemi *et al.* [23] used to. Here, 2 ml of 10 mM of sodium nitroprusside prepared in 0.5 mM phosphate buffer saline (pH 7.4) was added to 0.5 ml of different concentrations ranging from 0.025 to 0.5 mg/ml of each of plant extracts, BHT and gallic acid separately prepared,. Each of the mixtures were incubated at 25 °C for 150 min. This was followed by adding 0.5 ml of each incubated solution to 0.5 ml of Griess reagent containing 1.0 ml sulphanilic acid reagent (0.33% prepared in 20% glacial acetic acid at room temperature for 5 min with 1 ml of naphthylenediamine dichloride (0.1% w/v)). The mixture was incubated at room temperature for 30 min, followed by the measurement of the absorbance at 540 nm. The amount of nitric oxide radicals inhibited by the essential oil was calculated using Eq. (1):

(1) NO radical scavenging activity(%)=[(Abs control-Abs sample)/(Abs control)]×100

where Abs control is the absorbance of NO radicals + methanol; Abs sample is the absorbance of NO radical + extract or standard.

### 2.6 Ferric reducing power assay

The reducing power of the essential oil was determined by the method of Otang *et al.* [24] with slight modifications. Here, 0.5 ml (0.2 M) phosphate buffer (pH 6.6) and 0.5 ml of 0.1% potassium hexa-cyanoferrate were mixed with 0.5 ml of different concentrations of the essential oil ranging from 0.025-0.5 mg/ml before being incubate at 50 °C in a water bath for 20 min. After the incubation period, 0.5 ml of 10% trichloroacetic acid was added to terminate the reaction while 0.1 ml of 0.01% FeCl_3_ solution was added to 1 ml of the upper portion of the solution diluted with 1 ml of distilled water before the reaction mixture was being allowed to stand for 10 min at room temperature and the absorbance was measured at 700 nm against the appropriate blank solution. All tests were in triplicates. A higher absorbance of the reaction mixture indicated greater reducing power.

### 2.7 Hydrogen peroxide (H_2_O_2_) radical scavenging activity assay

The H_2_O_2_ inhibition activity of the essential oil was assessed by the method of Gülçin *et al.* [25]. Here, 0.6 ml of (4 mM) hydrogen peroxide solution prepared in phosphate buffer (0.1 M; pH 7.4) and incubated for 10 min was added to one ml (1 ml) of the essential oil, BHT and ascorbic acid at concentrations ranging from 0.025 to 0.5 mg/ml, respectively. The absorbance of the hydrogen peroxide at 230 nm was determined after 10 min against a blank solution containing phosphate buffer solution without hydrogen peroxide. The BHT and ascorbic were used as positive controls. The percentage scavenging of hydrogen peroxide of samples was calculated using Eq. (2):

(2) $${\text{H}}_{2}{\text{O}}_{2}\text{ inhibition capacity }\left(\text{\%}\right)=\left[1-\left({\text{H}}_{2}{\text{O}}_{2}\text{ cons. of sample}/{\text{H}}_{2}{\text{O}}_{2}\text{ cons. of blank}\right)\right]\times 100$$

### 2.8 Lipid peroxidation scavenging activity assay

An inhibition of lipid peroxidation in the rat liver homogenate was determined using a modified thiobarbituric acid reactive species (TBARS) assay described by Murugan and Parimelazhagan [26]. The liver homogenate (0.5 ml, 10% in distilled water (v/v)) and 0.1 ml of essential oil were mixed separately in a test tube and the volume was made up to 1 ml by adding distilled water. Finally, 0.05 ml of FeSO_4_ (0.07 M) was added to the mixture and incubated for 30 min to induce lipid peroxidation. This was followed by adding 1.5 ml of 20% acetic acid, 1.5 ml of 0.8% TBA (w/v) in 1.1% sodium dodecyl sulphate (SDS) and 0.05 ml of 20% TCA before vortexing and heating in a boiling water bath for 60 min. After cooling, 5.0 ml of butanol was added to each tube and centrifuged at 3000 rpm for 10 min. The absorbance of the organic upper layer was measured at 532 nm to quantify TBARS. For the blank, 1 ml of distilled water was used in place of the essential oil. Inhibition (%) of lipid peroxidation was calculated using Eq. (3):

(3) %Inhibition=[(Control OD-Sample OD)/Control OD]×100

### 2.9 DPPH radical scavenging activity assay

The DPPH free radical scavenging activity was determined with the method of Liyana-Pathiranan and Shahidi [27]. Here, 1 ml of 0.135 mM 2,2-diphenyl-1-picrylhydrazyl radical (DPPH) prepared in methanol was mixed with 1.0 ml of the essential oil, BHT and ascorbic acid at concentrations ranging from 0.025 to 0.5 mg/ml. The reaction mixture was then vortexed thoroughly and left in the dark at room temperature for 30 min. The absorbance of the mixture was measured using spectrophotometer at 517 nm. The actual decrease in absorbance was measured against that of the control. All tests and analysis were done in triplicates. The scavenging ability of the essential oil was then calculated using Eq. (4):

(4) DPPH scavenging activity(%)=[(Abs control-Abs sample)/(Abs control)]×100

where; Abs control is the absorbance of DPPH + methanol; Abs sample is the absorbance of DPPH radical + sample (sample or standard).

### 2.10 2, 2′-Aazino-bis (3-ethylbenzthiazoline-6-sulphonic acid) (ABTS) radical scavenging assay

The method described by Olajuyigbe and Afolayan [28, 29] was adopted for the determination of ABTS scavenging activity. Briefly, the stock solutions containing 7 mM ABTS solution and 2.4 mM potassium persulfate solution were prepared. The working solution was then prepared by mixing the two stock solutions in equal proportions and allowed to react for 12 h at room temperature in the dark. The solution was then diluted by mixing 1 ml ABTS^+^ solution with 60 ml of methanol to obtain an absorbance of 0.708 ± 0.001 units at 734 nm using the spectrophotometer. The essential oil (1 ml) and the controls were allowed to react with 1 ml of the ABTS^+^ solution and the absorbance was taken at 734 nm after 7 min with the spectrophotometer. The ABTS^+^ scavenging capacity of the extract was then compared with that of the standards. The percentage inhibition was then calculated using Eq. (5):

(5) Inhibition %=[(A blank-A sample)/A blank]×100

where A blank is the absorbance of ABTS radical + methanol used as control; A sample is the absorbance of ABTS radical + sample extract/standard. All the tests were carried out in triplicates. The activity was expressed as 50% inhibitory concentration (IC_50_). The lower the IC_50_ value, the higher the antioxidant activity.

## 3 Antibacterial analysis

### 3.1 Microorganisms and media

These bacterial strains used include *Salmonella typhi* ATCC 13311, *Enterococcus faecalis* ATCC 29212, *Escherichia coli* ATCC 25922, *Pseudomonas aeruginosa* ATCC 19582, *Bacillus cereus* ATCC10702, *Shigella sonnei* ATCC 29930, *Streptococcus pyogenes*, *Bacillus subtilis* KZN, *Shigella flexneri* KZN, *Vibrio cholerae* (Laboratory isolates), *Klebsiella pneumoniae* ATCC 4352 and *Staphylococcus aureus* ATCC 6538. The test organisms were obtained from the Department of Biochemistry and Microbiology, University of Fort Hare, South Africa. The bacterial isolates were maintained at 4 °C on agar plates and the inoculums for the assays were prepared by diluting scraped cell mass in 0.85% sodium chloride solution, adjusted to 0.5 McFarland standards and confirmed by spectrophotometric reading at 580 nm. The cell suspensions were finally diluted 1:100 in broth to give an approximate inoculum of 10^5^ cfu/ml as compared with McFarland standard used in the assays. The essential oil was prepared by being dissolved in 10% aqueous DMSO with Tween 80 (0.5% v/v for easy diffusion) before filtering through a 0.45 μm membrane filter [30].

### 3.2 In vitro antibacterial susceptibility assay by agar diffusion method

The agar well diffusion technique was employed as previously described by Prabuseenivasan *et al.* [30] and Otang *et al.* [31] with some modifications to determine the antibacterial susceptibility test. Briefly, 100 μl of 0.5 McFarland solutions of each bacterial culture in 0.85% sterile distilled water (SDW) was aseptically dispensed on the surface of sterile agar plates and spread evenly using sterile cotton swab to make a lawn. After inoculation, four wells were made in each agar plate with a heat sterilized 6 mm cork borer. Thereafter, 50 μl of amoxicillin (0.0125 mg/ml) used as the positive control and 50 μl of the essential oil were dispensed in the first and second wells respectively. In the third and fourth wells, 50 μl of a negative control which is the nutrient broth and the carrier vehicle (10% aqueous DMSO in Tween 80) were dispensed respectively. Each test was done in triplicate. The culture plates were then incubated at 37 °C and the results were observed after 24 h. The clear zones around each well indicating the activity of the essential oil against the bacterial isolates were measured in mm.

### 3.3 Gas chromatography mass spectrometry (GC/MS) analysis of the essential oil

GC-MS analyses were performed on Agilent 6890 Gas Chromatograph (GC) coupled to Agilent 5975 Mass Selective Detector (MSD) with a Zebron-5MS column (ZB-5MS 30 m × 0.25 mm x 0.25 um) (5%-phenylmethylpolysiloxane) (California, USA). The following column and temperature conditions were used: GC grade helium at a flow rate of 2 ml/min and splitless 1 ml injections was used. The injector, source and oven temperatures were set at 280 °C, 280 °C and 70 °C respectively. The ramp settings were 15 °C/min to 120 °C, then 10 °C/min to 180 °C, then 20 °C/min to 270 °C and held for 3 min.

### 3.4 Identification of components

The identification of the chemical constituents of the essential oil was determined by comparing their retention times, percentage composition (area %) and retention indices with those of authentic standards and their mass spectral fragmentation patterns (Willey/ChemStation data system) [32]. The identification was further confirmed by search using the National Institute of Standards and Technology (NIST) database (NIST/EPA/NIH mass spectral library (2014) with those of published data [33, 34]. Empirical searches were conducted using The PubChem Project (https://pubchem.ncbi.nlm.nih.gov/) and Drug Bank (www.drugbank.ca/) to identify the known pharmacological properties associated with these compounds.

### 3.5 Statistical analysis

All experiments were done in triplicates and the results were expressed as Mean ± SD. Where applicable, the data were subjected to one-way analysis of variance (ANOVA) followed by Duncan's Multiple Range test using the Minitab program (version 12 for windows). ρ < 0.05 were considered significant.

## 4 Results

**Figure 1:**
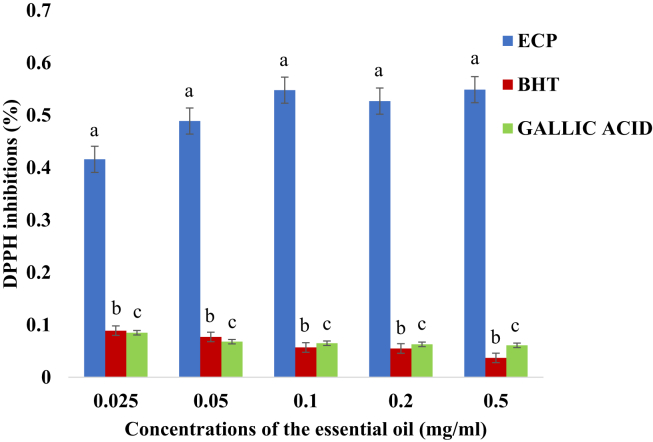
DPPH radical scavenging activity (%) (Mean + Standard deviation) of the essential oil of *E. caffra*. Key: ECP = Essential oil of *E. caffra*; BHT = Butylated hydroxyl toluene. Note: The average scavenging activities with different superscript at the same concentration of the essential oil are significantly different (ρ < 0.05).

In this study, there is an increasing trend in the antioxidant activity of the essential oil of *E. caffra* with respect to concentration. This shows that as the concentration of the essential oil increases, the DPPH radical scavenging activity of the essential oil increases significantly in comparison with those of BHT and Gallic acid (ρ < 0.05). However, reverse is the case in both butylated hydroxyl toluene (BHT) and gallic acid where both were decreasing as the concentration was increasing as shown in Figure 1.

**Figure 2:**
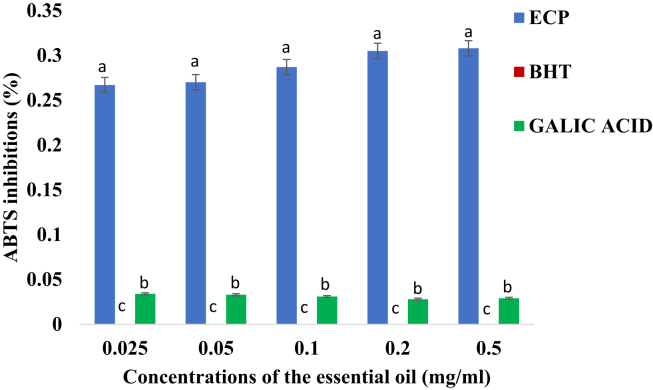
ABTS radical scavenging activity (%) (Mean + Standard deviation) of the essential oil of *E. caffra*. Key: ECP = Essential oil of *E. caffra*; BHT = Butylated hydroxyl toluene. Note: The average scavenging activities with different superscript at the same concentration of the essential oil are significantly different (ρ < 0.05).

Figure 2 shows a higher concentration dependent ABTS radical scavenging activity of the essential oil while those of both BHT and gallic acid indicated a constant value with a slight difference. Increase in concentrations indicated that both BHT and gallic acid were not significantly inhibited as the concentration of the essential oil increases. The essential oil was significantly more active against ABTS than the BHT and Gallic acid (ρ < 0.05).

**Figure 3:**
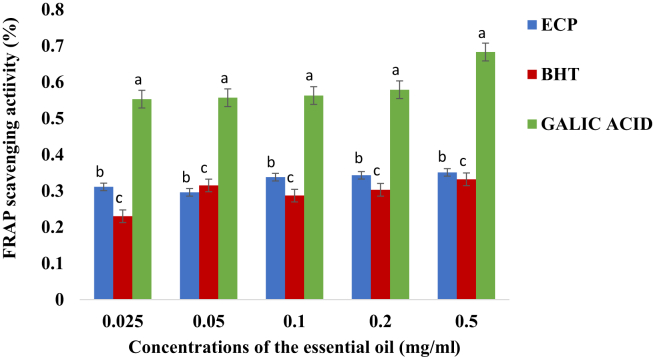
FRAP activity (%) (Mean + Standard deviation) of the essential oil of *E. caffra*. Key: ECP = Essential oil of *E. caffra*; BHT = Butylated hydroxyl toluene. Note: The average scavenging activities with different superscript on the same concentration of the essential oil are significantly different (ρ < 0.05).

In Figure 3, the ferric reducing antioxidant power (FRAP) of the ferric reducing antioxidant activity of the essential oil against BHT and gallic acid did not significantly change with increase in concentrations. The FRAP activity of the gallic acid was significantly higher than those of the BHT and the essential oil (ρ < 0.05).

**Figure 4:**
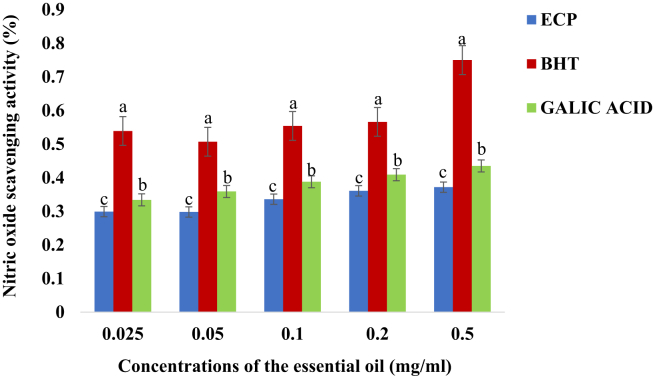
Nitric oxide scavenging activity (%) (Mean + Standard deviation) of the essential oil of *E. caffra.* Key: ECP = Essential oil of *E. caffra*; BHT = Butylated hydroxyl toluene. Note: The average scavenging activities with different superscript on the same concentration of the essential oil are significantly different (ρ < 0.05).

In Figure 4, the nitric oxide scavenging activity of the essential oil was concentration dependent. The nitric oxide scavenging activity of the BHT was significantly higher than those of the Gallic acid and the essential oil (ρ < 0.05). The concentration-dependent nitric oxide scavenging activity of the essential oil was the least of the recorded antioxidant activities.

**Figure 5:**
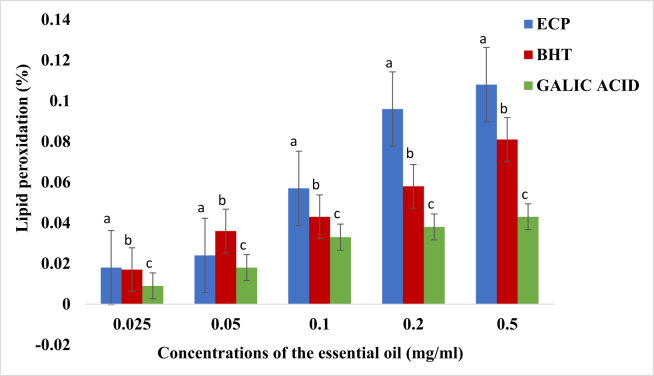
Lipid peroxidation activity (%) (Mean + Standard deviation) of the essential oil of *E. caffra*. Key: ECP = Essential oil of *E. caffra*; BHT = Butylated hydroxyl toluene. Note: The average scavenging activities with different superscript on the same concentration of the essential oil are significantly different (ρ < 0.05).

From the result obtained, the essential oil of *E. caffra* significantly inhibited the formation of TBARS in rat liver homogenate in a concentration dependent manner as shown in Figure 5. The percentage inhibition of lipid peroxidation in rat liver by the oil was significantly higher than those of BHT and Gallic acid (ρ < 0.05).

**Figure 6:**
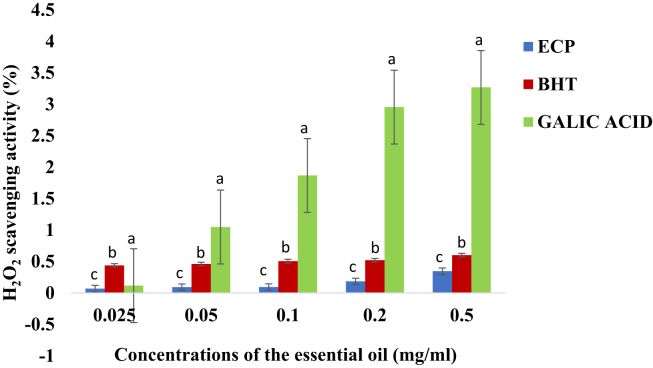
Hydrogen peroxide radical scavenging activity (%) (Mean + Standard deviation) of the essential oil of *E. caffra*. Key: ECP = Essential oil of *E. caffra*; BHT = Butylated hydroxyl toluene. Note: The average scavenging activities with different superscript on the same concentration of the essential oil are significantly different (ρ < 0.05).

When the activity of the essential oil on hydrogen peroxide was measured, it was observed that the essential oil was less active than those of BHT and Gallic acid used as control as shown in Figure 6. The activity of gallic acid on the hydrogen peroxide activity was significantly higher than those of the essential oil and BHT (ρ < 0.05). The activity of the essential oil was the least.

The susceptibility of the different test bacterial isolates to the activity of the essential oil and amoxicillin used as control was presented in Table 1. The susceptibility study showed that all bacterial isolates were susceptible to essential oil with the exception of *Salmonella typhimurium* and *Pseudomonas aeruginosa*. While the essential oil produced inhibition zones ranging between 22 ± 1.3 and 35 ± 2.1 mm in the susceptible isolates, amoxicillin produced inhibition zones ranging between 35 ± 2.1 and 40 ± 0.2 with the exception of *Pseudomonas aeruginosa* which is resistant to the antibacterial agents.

**Table 1:** Antibacterial activities of essential oil from *E. caffra* as compared to those amoxicillin (±1.0 mm).

Test bacterial isolates	Essential oil	Amoxicillin used as control
	Inhibition zones (±1.0 mm)	
*Salmonella typhimurium*	NA	36 ± 1.1
*Enterococcus faecalis*	25 ± 1.3	34 ± 1.2
*Escherichia coli*	22 ± 1.3	35 ± 0.2
*Pseudomonas aeruginosa*	NA	NA
*Bacillus cereus*	25 ± 1.3	32 ± 1.2
*Shigella sonnei*	25 ± 1.3	35 ± 2.2
*Streptococcus pyogenes*	30 ± 0.2	35 ± 2.1
*Bacillus subtilis*	28 ± 1.3	40 ± 0.2
*Shigella flexneri*	30 ± 0.2	40 ± 1.1
*Klebsiella pneumoniae*	25 ± 1.3	33 ± 2.2
*Staphylococcus aureus*	30 ± 0.2	37 ± 0.2
*Proteus vulgaris*	35 ± 2.1	38 ± 1.1
*Serratia marcescens*	35 ± 2.1	35 ± 2.2

**Figure 7:**
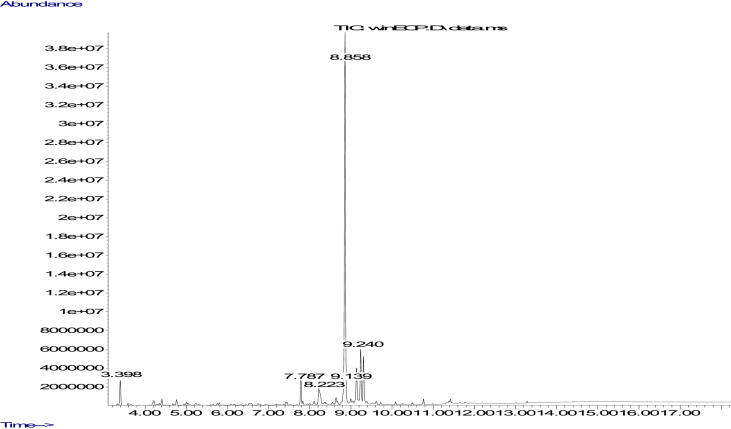
GC-MS chromatogram for essential oil of *Erythrina caffra.* Numbers in blue and red colours represents the compound's retention time and their corresponding identity in Table 2.

The GC-MS chromatogram for the bioactive compounds in the essential oil of *E. caffra* is presented in Figure 7 while the 35 compounds present in the essential oil are shown in Table 2. While the pharmacological activities of some of the identified compounds are indicated in Table 2, the pharmacological activities of some of the compounds are yet to be reported. Of these compounds, Caryophyllene oxide (53.54%), [1S-(1α,7α,8aβ)]-1,2,3,5,6,7,8,8a-octa-1 - hydro-1,8a-dimethyl-7-(1-methylethenyl)-Naphthalene (7.81%), Kauran-18-al (6.49%), 10,10-Dimethyl-2,6-dimethylenebicy clo[7.2.0]undecan-5β-ol (5.83%), 10s,11s-Himachala-3(12),4-diene (4.51%), Caryophyllene (3.65%) and 1- Hexanol (3.31%) are the most prominent compounds.

**Table 2:** Bioactive components of the essential oils of *Erythrina caffra* Thumb and their pharmacological activities.

S/N	RT	Name of the compound	Nature of compound	Molecular formula	Molecular Mass (g/mol)	Area (%)	Known pharmacological activity	References
1	3.398	1- Hexanol	Alcohol	C_6_H_14_O	102.17	3.31	Anesthetics, nicotinic antagonists Flavouring agent	Chorazewski *et al.* [35]
2	3.585	2-Heptanone	Ketone	C_7_H_14_O	114.19	0.23	Anaesthetic and Pesticide	Papachristoforou *et al.* [36]; Bilbao-Sáinz *et al.* [37]
3	4.213	Benzaldehyde	Aromatic Aldehyde	C_7_H_6_O	106.12	0.94	Anticancer and antifungal	Serda *et al.* [38]
4	4.407	2-pentyl- Furan	Aromatic cyclic ether	C_9_H_14_O	138.21	0.79	Flavouring agent	Chambers *et al.* [39]
5	4.766	D-Limonene	Terpene (monoterpene)	C_10_H_16_	136.23	0.83	Anti-stress and sedative properties, gastro esophageal reflux disease and heartburn	Park [40]; Zhou [41]; do Vale [42]; Sun [43]
6	5.007	1-Octanol	Fatty alcohol	C_8_H_18_O	130.23	0.32	Neurological tremors.	Bushara *et al.* [44]
7	5.044	3,5-Octadien-2-one	α,β,γ,δ – unsaturated ketone	C_10_H_16_	124.18	0.17	Sex pheromone	Zeeck *et al.* [45]; Gopalakrishnan and Udayakumar [46]
8	5.755	1,7,7-trimethylbicyclo[2.2.1] heptan-2-one (Camphor)	Monoterpene	C_10_H_16_O	152.23	0.25	Unknown	-
9	5.805	Nonyl chloroformate	Ester	C_10_H_19_ClO_2_	206.71	0.30	Unknown	**-**
10	6.528	5-methyl-2-(1-methylethyl)-2-Cyclohexen-1-one	Monoterpene	C_10_H_16_O	152.23	0.11	Unknown	-
11	7.424	Epizonarene	Sesquiterpene	C_15_H_24_	204.35	0.38	Unknown	http://www.naturalmedicinefacts.info/chemical-activities/1254.html - [47]accessed 11-8-2020
12	7.454	α-Copaene	Tricyclic sesquiterpenes	C_15_H_24_	204.35	0.28	Antioxidant, oxidative, cytotoxic, anti-genotoxic, cytogenetic effect	Türkez *et al.* [48, 49]
13	7.787	Caryophyllene	Bicyclic sesquiterpene	C_15_H_24_	204.35	3.65	Anti-inflammatory, anti-cancerous,local anesthetic, Antinociceptive, neuroprotective, anxiolytic, antidepressant and anti-alcoholism and Contributes to aroma of black pepper	Gertsch *et al.* [50]; Katsuyama *et al.* [51]; Guimarães-Santos [52]; Bahi *et al.* [53]; Shamma *et al.* [54]; Jirovetz *et al.* [55]
14	7.833	(+)-epi-Bicyclosesquiphellandrene	Sesquiterpene	C_15_H_24_	204.35	0.55	antidermatophytic activity, insecticidal activity	Kuiate *et al.* [56]; Urzúa *et al.* [57]
15	8.107	γ-Muurolene	Sesquiterpene	C_15_H_24_	204.35	0.76	Unknown	
16	8.223	10S,11S-Himachala-3(12),4-diene		C_15_H_24_	204.35	4.51	Unknown	
17	8.364	(1α,4aβ,8 aα)-1,2,3,4,4a,5,6,8a-oct ahydro-7-methyl-4-methylene-1-(1-m ethylethyl)Naphthalene	Sesquiterpene	C_15_H_24_	204.35	0.67	Unknown	
18	8.554	1,3,3-Trimethyl-2-hydroxymethyl-3, 3-dimethyl-4-(3-methylbut-2-enyl)-cyclohexene	Sesquiterpenoid	C_15_H_26_O	222.37	0.46	Unknown	
19	8.641	(Z)-3-methyl-2-pentenyl-2-cyclopenten-1-one	Ketone or cyclic α,β unsaturated ketone	C_11_H_16_O	164.24	1.11	Unknown	
20	8.677	Aristolene epoxide	Sesquiterpenoid	C_15_H_24_O	220.35	0.50	Unknown	
21	8.858	Caryophyllene oxide	Sesquiterpenoid	C_15_H_24_O	220.35	53.54	Antioxidant, anti-inflammatory and antibacterial, use in Fragrances and Flavors industries.	Raja *et al.* [58]; Mihailović *et al.* [59]; Raman *et al.* [60]
22	8.999	([4aR]-4aα,7α,8aβ)-decahydro-4a-methyl-1 -methylene-7-(1-methylethenyl)-Naphthalene	Sesquiterpene	C_15_H_24_	204.35	1.15	Unknown	
23	9.055	(1α,3aβ,4α,7aβ)]-octahydro-1,7a-dimethyl-4-(1-methylethenyl)-1,4-methano-1H-indene	Sesquiterpene	C_15_H_24_	204.35	0.70	Unknown	
24	9.139	10,10-Dimethyl-2,6-dimethylenebicy clo[7.2.0]undecan-5β-ol	Sesquiterpenoid	C_15_H_24_	220.35	5.83	Unknown	
25	9.183	[4aR]-(4aα,7α,8aβ)-decahydro-4a-methyl-1 -methylene-7-(1-methylethenyl)-Naphthalene	Sesquiterpene	C_15_H_24_	204.35	0.44	Unknown	
26	9.240	[1S-(1α,7α,8aβ)]-1,2,3,5,6,7,8,8a-octa-1 - hydro-1,8a-dimethyl-7-(1-methylethenyl)-Naphthalene	Sesquiterpene	C_10_H_16_	204.35	7.81	Unknown	
27	9.308	Kauran-18-al	Diterpenoid	C_21_H_34_O_3_	302.5	6.49	Unknown	
28	9.618	5,5-dimethy l-4-(3-methyl-1,3-butadienyl)-1-Oxaspiro[2.5]octane	Sesquiterpene derivative	C_14_H_22_O	206.32	0.55	Unknown	
29	9.724	1-Pentadecene	Alkene	C_15_H_30_	210.4	0.24	Unknown	**-**
30	10.087	6,10,14-trimethyl 2-Pentadecanone	Ketone or metabolized diterpene	C_18_H_36_O	268.48	0.36	Unknown	
31	10.499	6,10,14-trimethyl-, (E,E)-5,9,13-Pentadecatrien-2-one	Ketone or metabolized diterpene or phytol derivative	C_18_H_30_O	262.42	0.35	Unknown	
32	10.767	Nabumetone	Naphthalene derivative	C_15_H_16_O_2_	228.29	0.76	Nonsteroidal anti-inflammatory drug (NSAID)	MedPlus [61]
33	11.384	3-amin o-5-(diethylamino)-2,6-Pyrazinedicarbonitrile	Pyrazine derivative	C_10_H_12_N_6_	216.24	0.36	Unknown	
34	11.419	1-methoxy-1,9,12-Octadecatriene	Sesquiterpene oxide	C_19_H_34_O	278.5	0.36	Unknown	
35	13.283	Diisooctyl phthalate	Aromatic ester	C_24_H_38_O_4_	390.6	0.20	Unknown	

## 5 Discussion

Overproduction of free radicals or reactive oxygen species (ROS) causes oxidative stress and disease [62, 63, 64, 65]. Free radicals and reactive oxygen species have been shown to cause oxidative damages on biomolecules including proteins, amino acids, lipids and DNA [66] and its excessive production has been linked with several diseases [67]. Consequently, oxidative stress resulting from imbalance between excessive generation of free radicals and inadequate antioxidant defense system has been linked to pathogenesis of many diseases such as diabetes, cancer, atherosclerosis and inflammation [68, 69]. In addition to this, synthetic antioxidants such as butylated hydroxyanisole (BHA), butylhydroxytoluene (BHT) or propyl gallate, largely used as food additives, have been reported to be injurious to the human body [70]. Therefore, while there is a need to enhance the antioxidant system in order to regain the balance, increased use of naturally occurring antioxidants has been considerably regarded as an effective and safe way to prevent ROS-induced diseases. While medicinal plants have been used in folk medicine for the treatment of numerous diseases, essential oil and various extracts from plant, for many decades, have been studied for their natural products, efficiency and effectiveness in the treatment of infectious and dreadful diseases [71, 72]. Since they possess compounds having protective effects against these diseases, essential oils and phenolic compounds of plant origin have been highly investigated by researchers for their antioxidant properties [70, 73]. Thus, polyphenolic compounds including flavonoids suppress reactive oxygen formation, chelate trace elements involved in free-radical production, scavenge reactive species and up-regulate and protect antioxidant defenses [74] to contribute directly to antioxidant capacity and exhibit their protective functions against oxidative damage and health benefits [75, 76, 77].

In this study, the antioxidant activities of the essential oil of *Erythrina caffra* determined were higher and concentration-dependent moderate inhibitions in nitric oxide, reducing power, hydrogen peroxide, lipid peroxidation inhibitory assays, DPPH, and ABTS scavenging activity assays. While antioxidant efficiency is often associated with the ability of an extract to scavenge stable free radicals, this essential oil possessed the capability to scavenge the hydroxyl radical, superoxide radical, hydrogen peroxide, singlet oxygen and nitric monoxide produced with strong antioxidant activity better than the BHT and gallic acid used as control. That the essential oil of *E. caffra* showed a concentration dependent activity against reactive oxygen species indicated that its administration could significantly increases superoxide dismutase which is the first antioxidant defense in an oxidative stress situation [78] and catalase which catalyzes the decomposition of hydrogen peroxide to water and molecular oxygen [79, 80] in a dose dependent manner. On the other hand, since the power of antioxidant activity to reduce ferric ion indicated the potential antioxidant activity, the exponential rise in antioxidant activity as a function of the reducing ability showed that antioxidant potentials are linked with reducing power [81]. Thus, while essential oils have therapeutic uses in human medicine due to its anticancer, antinociceptive, antibacterial and antioxidant properties [82, 83], they act as chain-breaking antioxidants to scavenge and destroy reactive oxygen species (ROS) [84].

Unsaturated lipids in liver tissue are very predisposed to peroxidation when exposed to reactive oxygen species (ROS) or free radicals and the most predisposed ones are the polyunsaturated fattyacids. Stark [85] and Ott *et al.* [86] reported that overproduction of reactive oxygen species readily attacks the polyunsaturated fatty acids in the plasma membrane leading to oxidative degradation of lipids. Birben *et al.* [87] indicated that the lipid oxidation induces cellular and tissue damages through covalent bonding resulting in lipid peroxidation, DNA injury, inflammation and subsequent cell death. St. Angelo *et al.* [88] and Gülçin [89] report that lipid oxidation in foods and food products lowers food quality, creating off-flavors and unhealthful compounds. It, therefore, implies that increased lipid peroxidation weakens membrane function by reducing membrane fluidity and changing the activity of membrane-bound enzymes and receptor [90]. This study showed that *E. caffra* essential oil prevented lipid peroxidation activity at the lowest concentration when compared to gallic acid and BHT used as the controls. The essential oil could have prevented lipid peroxidation by mopping up ROS, prevented peroxyl radical formation and disable the radical from attacking another fatty acid to form lipid hydroperoxide. Thus, by preventing ROS production and lipid oxidation, the antioxidant activity of this essential oil may preserve foods from the toxic effects of oxidants [91].

The result of antimicrobial activity of the essential oil of *E. caffra* revealed that the oil possess good antimicrobial activity against Gram positive and Gram negative bacteria. The test bacterial isolates were susceptible to the essential oil with the exception of *Salmonella typhimurium* and *Pseudomonas aeruginosa*. In addition, the antibacterial activity could be attributed to the presence of caryophyllene oxide, a sesquiterpene oxide, which is predominant in the oil while the contribution of [1S-(1α,7α,8aβ)]-1,2,3,5,6,7,8,8a-octa-1 - hydro-1,8a-dimethyl-7-(1-methylethenyl)-Naphthalene (7.81%), Kauran-18-al (6.49%) and 10,10-Dimethyl-2,6-dimethylenebicy clo[7.2.0]undecan-5α-ol (5.83%) with unknown biological activities may not be underestimated. While sesquiterpenes and monoterpenes possess antimicrobial activity against Gram-positive and Gram-negative human pathogens [92], the biological activity of essential oil of *E. caffra* may be attributed to the presence of certain alcohols, ethers, ketone, and aldehyde identified as bioactive compounds from the GC-MS analysis of the extract. An important characteristic of essential oils and their constituents is their hydrophobicity which enables them to partition in the lipids of bacterial cell membrane and mitochondria thus disturbing the structures and rendering them more permeable [93]. This can lead to leakages of ions and other molecules in the cell and consequently, resulting in cell death [94, 95, 96].

## 6 Conclusion

The new interest of the pharmaceutical industries in essential oils as possible natural substances to replace synthetic drugs and preservatives has redirected the focus of many researchers. This study revealed that the essential oil of *E. caffra* stem bark extract possess antimicrobial and good antioxidant activities. Since antioxidants play a major role in managing some free radical-related diseases like cancers, the essential oil of *Erythrina caffra* with its rich level of phytochemicals can be used as either dietary or complementary agents. In addition, the oil may be explored for the development of useful plant-based pharmaceuticals, food preservatives and as carriers of other additives such as flavor in processed foods and fragrance in cosmetic production. Furthermore, *in vivo* studies may be carried out to reconfirm the lipid lowering activity of the essential oil.

## Declarations

### Author contribution statement

Olubunmi A. Wintola; Olufunmiso O. Olajuyigbe: Conceived and designed the experiments; Performed the experiments; Analyzed and interpreted the data; Contributed reagents, materials, analysis tools or data; Wrote the paper.

Aderonke A. Olajuyigbe: Performed the experiments; Analyzed and interpreted the data; Wrote the paper.

Anthony J. Afolayan: Contributed reagents, materials, analysis tools or data; Wrote the paper.

Roger M. Coopoosamy: Analyzed and interpreted the data; Contributed reagents, materials, analysis tools or data; Wrote the paper.

### Funding statement

This research did not receive any specific grant from funding agencies in the public, commercial, or not-for-profit sectors.

### Data availability statement

Data included in article/supplementary material/referenced in article.

### Declaration of interests statement

The authors declare no conflict of interest.

### Additional information

No additional information is available for this paper.
